# Editorial: Nonlinear dynamics and complex patterns in the human musculoskeletal system and movement

**DOI:** 10.3389/fbioe.2023.1339376

**Published:** 2023-12-14

**Authors:** Yih-Kuen Jan, Cheng-Feng Lin, Fuyuan Liao, Navrag B. Singh

**Affiliations:** ^1^ Rehabilitation Engineering Lab, Department of Kinesiology and Community Health, University of Illinois at Urbana-Champaign, Champaign, IL, United States; ^2^ Department of Physical Therapy, National Cheng Kung University, Tainan, Taiwan; ^3^ Department of Biomedical Engineering, Xi’an Technological University, Xi’an, China; ^4^ Department of Health Sciences and Technology, ETH Zurich, Zurich, Switzerland; ^5^ Singapore-ETH Centre, Future Health Technologies Program, CREATE Campus, Singapore, Singapore

**Keywords:** complexity, dynamics, modeling, movement, nonlinearity, tissue

The human body consists of interacting systems, such as musculoskeletal, nervous, and cardiovascular systems, that work together to perform complex body functions and movements (Shiogai et al., 2010; Stergiou and Decker, 2011). In the past decades, researchers have focused on the study of individual systems using biomechanical approaches to better understand the structure and function of the human system and how pathological diseases affect the human musculoskeletal system function and movement (Jan et al., 2013; Liao and Jan, 2017; Liau et al., 2019). Although the concepts of non-linear dynamics and complex patterns have been recognized as emerging methods to better understand the human musculoskeletal system and movement, the non-linear dynamics and complex patterns of human musculoskeletal system and movement remain largely unexplored (Shiogai et al., 2010; Stergiou and Decker, 2011; Orter et al., 2019). This Research Topic aims to highlight novel applications of non-linear dynamic and complexity methods to understand the human musculoskeletal system and its interactive systems (e.g., nervous, cardiovascular, and integumentary systems) and movement in healthy and pathological populations.

Concepts of non-linear dynamics and complex interactions in the human musculoskeletal system and movement refer to variability, adaptability, and pattern formation. Wang et al. investigated the interaction between changes in muscle activation and cortical network dynamics during isometric elbow contraction using surface electromyography (sEMG) and functional near-infrared spectroscopy (fNIRS). Fuzzy approximate entropy was used to evaluate signal complexity in various motor tasks. Their results demonstrated that connectivity between brain regions assessed through fNIRS in motor tasks on the dominant side was significantly higher and that there was a significant positive correlation between entropy and hemodynamic values in the contralateral brain regions in both dominant and non-dominant sides. These findings provide evidence of the interaction between the brain and the musculoskeletal system. Wolff et al. explored the effects of age, body dimension, and handgrip strength on the plantar pressure trajectory. Their results indicate that age, body weight, body height, and body mass index can explain up to 46% of the variability in plantar pressure trajectories. They conclude that age and body dimension should be used to adjust plantar pressure data. Burdack et al. intended to detect cross-movement commonalities of individual walking, running, and handwriting patterns using data augmentation. They used the conditional cycle-consistent generative adversarial network (CycleGAN). The model was trained by data obtained from 17 healthy adults and was then used to artificially generate other movement data. The classification F1-score ranged from 46.8% for handwriting data and 98.9% for walking data. Their model supports the existence of inter-individual differences while performing various activities, with handwriting being more personalized than walking or running.

In this Research Topic, studies explored the interactions between components of the musculoskeletal system and their effect on movement performance. Bai et al. explored the use of non-negative matrix factorization (NNMF) to extract mechanical characteristics of foot functional units during walking and running. Their results demonstrate that walking and running can be decomposed into two foot functional units, namely, buffering and push-off. The forefoot occupies a certain weight in both buffering and push-off functions indicating that there may be a complex foot function transformation mechanism in the transverse arch of the foot. Their study suggests that NNMF is feasible for analyzing foot mechanical characteristics. Moon et al. used a musculoskeletal modeling approach to investigate the effect of an increase in sprinting velocity on the load of the anterior cruciate ligament and knee joint. Their results indicate that factors such as knee joint shear force, extended landing posture with increasing sprinting velocity, internal rotation moment, and femoral muscle activity imbalance influence the increase of anterior cruciate ligament load during a sidestep cutting maneuver. He et al. used a musculoskeletal modeling approach to investigate the differences in lumbar and pelvis movements between cross-court and long-line topspin forehand strokes in table tennis. They found that the lumbar and pelvis embody greater weight transfer and greater energy production mechanisms in cross-court compared to long-line topspin forehand. Xi et al. intended to address soft tissue artifacts when quantifying the motion of the spine using optical motion capture with retroreflective markers. They found that anatomical direction, marker location, and anatomical segment depend on soft tissue artifacts. These findings could help develop location- and direction-specific weighting factors for use in global optimization algorithms aimed at minimizing the effects of soft tissue artifacts on the calculation of lumbar joint kinematics.

Because human systems are complex, the use of linear and non-complex methods might not allow a comprehensive understanding of how pathological diseases, as well as aging and growth, affect the performance of the human musculoskeletal system and movement. In this Research Topic, finite element modeling has been used to quantify these interactions. Wang et al. evaluated the effectiveness of an innovative hourglass-shaped graft *versus* a traditional columnar graft for restoring joint stability and avoiding notch impingement following anterior cruciate ligament reconstruction. Using finite element modeling, the authors found that with a knee flexion angle of 30°, an hourglass-shaped graft was better able to restore joint stability compared to columnar grafts. Another finite element simulation was conducted by Wan et al. to investigate the cause of polyethylene liner dissociation after reverse shoulder arthroplasty. Their results demonstrate that humeral adduction impingement could lead to the deformation of the claw-shaped liner fixation structure, which might be one of the reasons for the linear dissociation, and the increased stiffness of the liner material helps to reduce the deformation of the fixation structure. Hsieh et al. proposed a non-articulating, additively manufactured hybrid total disc replacement (TDR) with an ultra-high molecular weight polyethylene core and polycarbonate urethane fiber jacket to mimic the motion of normal discs. A finite element study was conducted to optimize the lattice structure and assess the biomechanical performance of the proposed TDR. Their finding suggests the feasibility of implanting an additively manufactured multi-material artificial disc that allows for better physiological motion than the current ball-and-socket design. Wei et al. investigated the mechanical properties of tibial plateau fractures after internal fixation and bone using a finite element model. Their results indicate that the internal fixation system tolerates some of the body’s typical actions and may sustain all or part of the weight early in the postoperative period.

This Research Topic also highlights novel applications of non-linear dynamic and complexity methods in assessing the musculoskeletal system and movement in various pathophysiological conditions. Xv et al. used the common chimpanzee to elucidate the evolution mechanisms of hominin bipedality by exploring the effect of skeletal architecture and muscle properties on bipedal standing. The authors proposed a musculoskeletal model with the mechanical relationships of the Hill-type muscle-tendon units. Their finding bridges the gap between skeletal architecture, along with muscle properties, and the biomechanical performance of the common chimpanzee during bipedal standing, which advances the understanding of bipedal evolution in humans. Luis et al. compared estimated muscle excitation and muscle fiber lengths among model selection, and performance criteria to solve muscle redundancy and approaches for scaling muscle-tendon properties at various walking speeds. They found that there were discrepancies in estimating fiber lengths and muscle excitations among the models and no single model combination estimated the most accurate muscle excitations for all muscles.

It is evident from the articles in this Research Topic that non-linear and complex properties and their interactions exist in the human movement and musculoskeletal system. The Research Topic of articles available within this Research Topic provides the foundation for further communication between clinical and non-linear dynamics and complexity researchers to advance our understanding of complex human musculoskeletal systems and related systems and movement for making better clinical decisions on assessments and treatments. Our Research Topic provides novel insights into the effects of various pathophysiological mechanisms, aging, and growth as well as rehabilitative interventions on the musculoskeletal system and movement, and thereby opens up new avenues for improved diagnosis and treatment of conditions of the musculoskeletal system.
